# Syntheses and structures of ammonium transition-metal dialuminium tris­(phosphate) dihydrates (NH_4_)*M*Al_4_(PO_4_)_3_·2H_2_O (*M* = Mn and Ni)

**DOI:** 10.1107/S2056989023000555

**Published:** 2023-01-26

**Authors:** Makoto Tokuda, Keita Tanaka, Kazumasa Sugiyama

**Affiliations:** a Institute for Materials Research, Tohoku University, 2-1-1 Katahira, Aoba-ku, Sendai 980-8577, Japan; University of Aberdeen, United Kingdom

**Keywords:** single-crystal diffraction, crystal structure, aluminophosphate

## Abstract

The structures of ammonium manganese(II) dialuminium tris­(phosphate) dihydrate, (NH_4_)MnAl_2_(PO_4_)_3_·2H_2_O, and ammonium nickel(II) dialuminium tris­(phosphate) dihydrate, (NH_4_)NiAl_2_(PO_4_)_3_·2H_2_O, were determined using single-crystal diffraction data. The structures of title compounds are isotypic to cobalt aluminophosphate, (NH_4_)CoAl_2_(PO_4_)_3_·2H_2_O (LMU-3) [Panz *et al.* (1998 ▸). *Inorg. Chim. Acta*, **269**, 73–82], in which a three-dimensional network of vertex-sharing AlO_5_ and PO_4_ moieties delineate twelve-membered channels in which ammonium, NH_4_^+^, and transition-metal cations (*M* = Mn^2+^ and Ni^2+^) reside as charge compensators for the anionic [Al_2_(PO_4_)_3_]^3–^ aluminophosphate framework. In both structures, the N atom of the ammonium cation, the transition-metal ion and one of the P atoms lie on crystallographic twofold axes.

## Abstract 2

The aluminophosphate frameworks of the title compounds, (NH_4_)*M*Al_2_(PO_4_)_3_·2H_2_O (*M* = Mn and Ni), consist of a three-dimensional array of vertex-sharing tetra­hedral PO_4_ and trigonal–bipyramidal AlO_5_ moieties, which delineate [001] twelve-membered channels in which the ammonium NH_4_^+^ and transition-metal cations (*M* = Mn^2+^ and Ni^2+^) reside as charge compensators for the anionic aluminophosphate framework.

## 1. Chemical context

The mixed-metal phosphate composed of tetra­hedral, bipyramidal and octa­hedral building units with chemical formula KNiAl_2_(PO_4_)_3_·2H_2_O was firstly reported by Meyer & Haushalter (1994 ▸). Isotypic structures were found in the alumino-, ferri- and gallophosphates; (NH_4_)CoAl_2_(PO_4_)_3_·2H_2_O (Panz *et al.* 1998 ▸), KMnAl_2_(PO_4_)_3_·2H_2_O (Kiriukhina *et al.* 2020 ▸), CsFe_3_(PO_4_)_3_·2H_2_O (Lii & Huang 1995 ▸), (NH_4_)CoGa_2_(PO_4_)_3_·2H_2_O (Chippindale *et al.* 1996 ▸), (NH_4_)MnGa_2_(PO_4_)_3_·2H_2_O (Chippindale *et al.* 1998 ▸), (NH_4_)NiGa_2_(PO_4_)_3_·2H_2_O (Bieniok *et al.* 2008 ▸) and KNiGa_2_(PO_4_)_3_·2H_2_O (Chippindale *et al.* 2009 ▸).

Herein, we report the syntheses and structures of (NH_4_)*M*Al_2_(PO_4_)_3_·2H_2_O [*M* = Mn in (I) and Ni in (II)] using a hydro­thermal technique and structural analysis by single-crystal X-ray diffraction. These compounds are isotypic to (NH_4_)CoAl_2_ (PO_4_)_3_·2H_2_O (LMU-3), crystallizing from a hydro­thermal synthesis (Panz *et al.*, 1998 ▸).

## 2. Structural commentary

**Figure 1:**
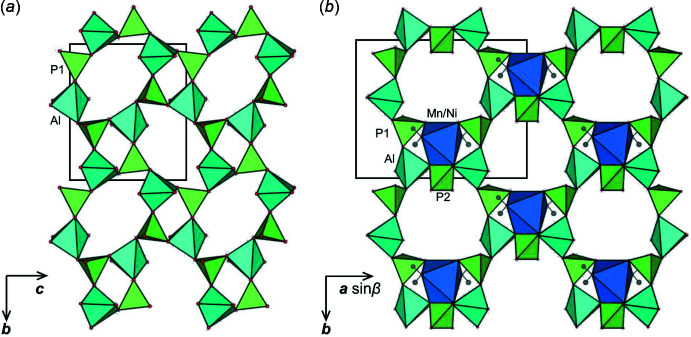
(*a*) Two-dimensional layer formed by four- and eight-membered rings in the *bc* plane and (*b*) the three-dimensional channels formed by twelve-membered rings in the aluminophosphate framework of Al_2_ *M*(NH_4_)(PO_4_)_3_·2H_2_O (*M* = Mn and Ni) illustrated using *VESTA* (Momma & Izumi, 2011 ▸).

The aluminophosphate framework of the title compounds with the chemical formula (NH_4_)*M*Al_2_(PO_4_)_3_·2H_2_O (*M* = Mn and Ni) is composed of [PO_4_] tetra­hedra and [AlO_5_] trigonal-bipyramids. Fig. 1 ▸(*a*) shows the [Al_2_(PO_4_)_3_]_∞_ layers, which are built up from four- and eight-membered rings connected *via* Al—O—P bonds. These layers stack along the *a*-axis direction, with the [P2O_4_] tetra­hedra (atom P2 lies on a crystallographic twofold axis) bridging between them, leading to the formation of a three-dimensional network encapsulating twelve-membered channels propagating in the [001] direction. The ammonium and transition-metal cations are respectively located in and on these channels, compensating the negative charge of the aluminophosphate framework [Fig. 1 ▸(*b*)].

There are two axial and three equatorial Al—O bonds within the [AlO_5_] trigonal bipyramids (Table 1 ▸). The axial Al—O bond distances for *M* = Mn are 1.8886 (19) and 1.9320 (18) Å and those for Ni are 1.8818 (14) and 1.9271 (14) Å, and the equatorial ones are in the ranges 1.7847 (19)–1.8080 (18) Å (Mn) and 1.7731 (14)–1.7979 (14) Å (Ni), thus the average axial Al—O bond distances are larger than the equatorial ones. Previous studies on [AlO_5_] trigonal bipyramids in LMU-3, KNiAl_2_(PO_4_)_3_·2H_2_O, KMnAl_2_(PO_4_)_3_·2H_2_O and (NH_4_)_3_Al_2_(PO_4_)_3_ (Panz *et al.*, 1998 ▸; Meyer & Haushalter 1994 ▸; Kiriukhina *et al.*, 2020 ▸; Medina *et al.* 2004 ▸) showed similar geometrical features with longer axial Al—O bonds distances.

**Figure 2:**
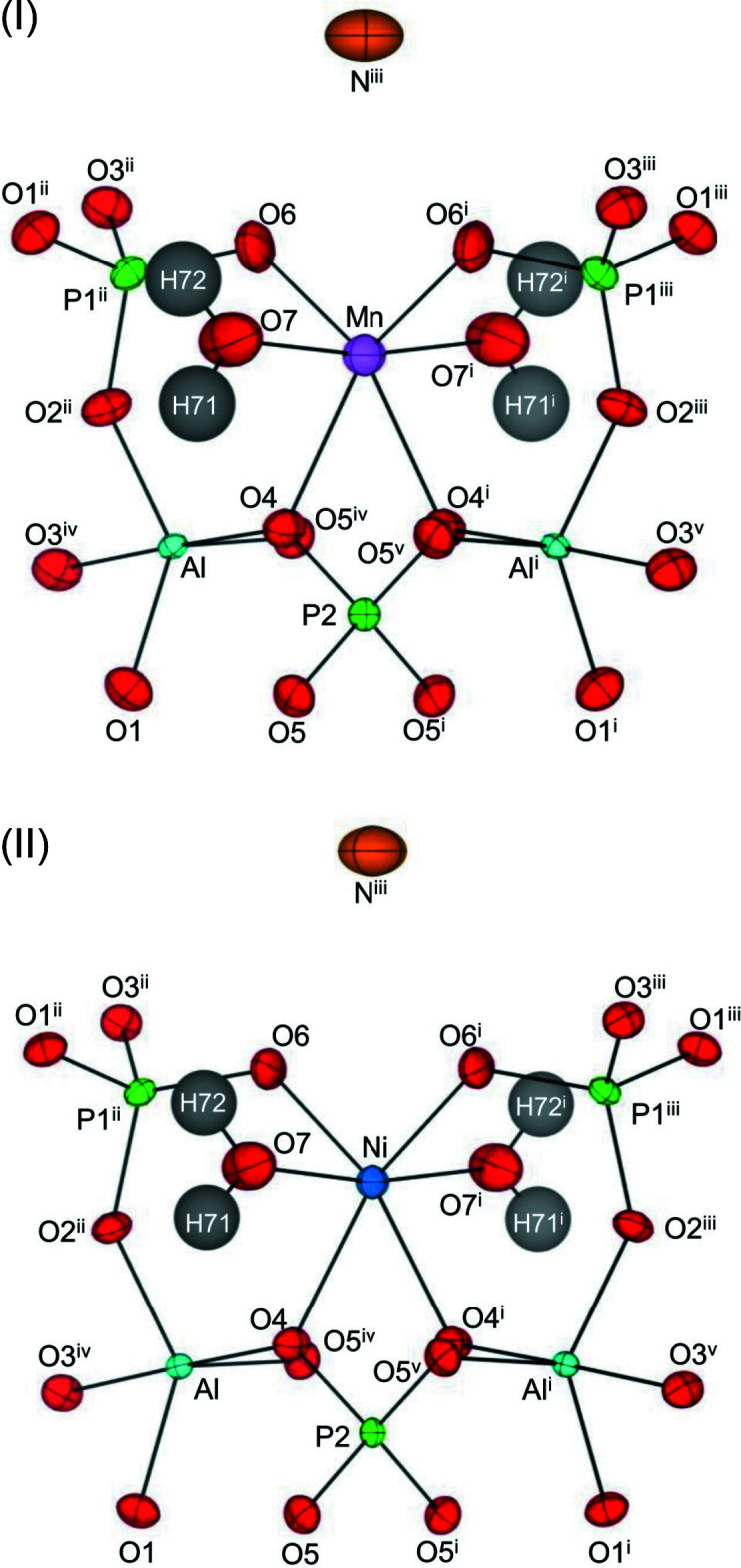
Positions of the *M*O_6_ [*M* = Mn (I) and Ni (II)] octa­hedra in the twelve-membered-ring channel of the aluminophosphate framework. Displacement ellipsoids are presented at the 80% probability level. [Symmetry codes: (i) −*x*, *y*, −*z* + 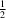 ; (ii) −*x* + 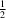 , *y* − 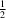 , −*z* + 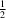 ; (iii) *x* − 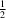 , *y* − 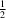 , *z*; (iv) *x*, −*y* + 1, *z* − 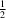 ; (v) −*x*, −*y* + 1, −*z* + 1.]

The transition-metal cations, which lie on crystallographic twofold axes, are octa­hedrally coordinated by two oxygen atoms of water mol­ecules and four oxygen atoms of the framework (Fig. 2 ▸). The mean *M*—O bond distances for the Mn and Ni compounds are 2.186 Å and 2.079 Å, respectively, which are consistent with the ionic radii of ^VI^Mn^2+^ (0.83 Å) and ^VI^Ni^2+^ (0.69 Å; Shannon 1976 ▸). The *M*O_6_ octa­hedron shares an edge O4⋯O4 with the adjacent [P2O_4_] tetra­hedron. The length of the shared-edge O4⋯O4 is the shortest among the twelve edges of octa­hedrally coordinated transition-metal cations in accordance with the P^5+^–*M* ^2+^ cation repulsion (Pauling, 1929 ▸, 1960 ▸).

The positions of the hydrogen atoms in the water mol­ecule, H71 and H72, could be determined by analysing the residual peaks in the difference-Fourier maps. The oxygen atom O7 of the water mol­ecule is coordinated to the transition-metal ions, and hydrogen atoms of H71 and H72 form O—H⋯O hydrogen bonds with the oxygen atoms O1 and O3 of the [Al_2_(PO_4_)_3_]_∞_ layer, respectively (Tables 2 ▸ and 3 ▸). Thus, the H71⋯O1 and H72⋯O3 hydrogen bonds contribute to the accumulation of the layers.

As for the hydrogen-bonding inter­actions of the ammonium cation (N atom site symmetry 2) within the title compounds, not all the H atoms could be definitively located from difference maps but some structural information could be obtained from the observed distances N1⋯O5 = 3.085 (5) and 3.103 (4) Å and N1⋯O6 = 2.906 (4) and 2.862 (3) Å for (NH_4_)*M*Al_2_(PO_4_)_3_·2H_2_O (*M* = Mn and Ni), respectively. The longer N1⋯O5 distance and the large isotropic atomic displacement parameters, *U*_iso_, of the N1 atom clearly indicate the relatively weaker hydrogen bonding for the presumed N1—H⋯O5 cases. This structural feature did not allow us to definitively located the positions of hydrogen atoms within the N1—H⋯O5 cases. Nevertheless, some of the hydrogen-atom positions around the ammonium cations could be located in the difference-Fourier maps and coordinates are (0.5382, 0.3998, 0.2391) and (0.5357, 0.4204, 0.2296) for (NH_4_)*M*Al_2_(PO_4_)_3_·2H_2_O (*M* = Mn and Ni), respectively. These possible hydrogen-atom positions correspond to those for the N1—H⋯O6 cases. Weak hydrogen bonds between NH_4_^+^ and the framework suggests that NH_4_^+^ and a monovalent cation (*e.g*., alkali cation or H_3_O^+^) are exchangeable akin to zeolitic cations in this unique framework structure (Meyer & Haushalter 1994 ▸; Kiriukhina *et al.*, 2020 ▸). The chemical formula for the group of compounds reported in this study can be denoted by *A* ^+^ *M* ^2+^Al_2_(PO_4_)_3_·2H_2_O (*A* = monovalent cation, *M* = divalent transition-metal cation).

## 3. Synthesis and crystallization

Single crystals of (NH_4_)*M*Al_2_(PO_4_)_3_·2H_2_O (*M* = Mn and Ni) were obtained as by-products of the laumontite-type zeolite imidazole-templated hydro­thermal technique. The precursor solution was prepared by dissolving the chemical agents of imidazole, aluminium-isopropoxide and H_3_PO_4_ (85% solution): the transition-metal component (Ni or Mn) was added to the solution. For the insertion of nickel in the system, (CH_3_COO)_2_Ni·4H_2_O was used and for corresponding manganese analogue (CH_3_COO)_2_Mn·4H_2_O was added to the as-prepared precursor solution. In each case, the resultant gel mixture was then sealed in a Teflon-lined tube and heated at 453 K for three days.

A few colorless, transparent crystals of (NH_4_)MnAl_2_(PO_4_)_3_·2H_2_O with a plate-like form were separated from the microcrystalline material together with the laumontite-type aluminophosphate, Mn-hureaulite Mn_5_[PO_3_(OH)]_2_(PO_4_)_2_·4H_2_O. In the case of Ni, the product comprises NH_4_NiAl_2_(PO_4_)_3_·2H_2_O, which forms colorless, transparent plate-like crystals and organic compounds.

The chemical analyses of the synthesized products were performed using energy-dispersive X-ray spectroscopy (EDS). The EDS profile clearly showed the presence of nitro­gen. This supports the idea that NH_4_^+^, a decomposition product of imidazole, was incorporated within the framework as a charge-compensating cation.

## 4. Refinement details

The crystal data, data collection methods, and structure refinement details are summarized in Table 4 ▸. The positions of the hydrogen atoms bonded to O7 were estimated using the residual peaks in the difference Fourier maps and refined using a riding model. The *U*_iso_ parameters for hydrogen atoms were fixed at 1.5 × the *U*_iso_ of O7.

## Supplementary Material

Crystal structure: contains datablock(s) global, I, II. DOI: 10.1107/S2056989023000555/hb8032sup1.cif

Structure factors: contains datablock(s) I. DOI: 10.1107/S2056989023000555/hb8032Isup2.hkl

Structure factors: contains datablock(s) II. DOI: 10.1107/S2056989023000555/hb8032IIsup3.hkl

CCDC references: 2237562, 2237561

Additional supporting information:  crystallographic information; 3D view; checkCIF report

## Figures and Tables

**Table 1:** Selected bond lengths (Å) in (NH_4_)*M*Al_2_(PO_4_)_3_·2H_2_O [*M* = Mn (I) and Ni (II)]

	(I)	(II)
*PO_4_ tetra­hedra*		
P1—O6^iv^	1.5152 (19)	1.5180 (14)
P1—O2	1.5342 (19)	1.5361 (14)
P1—O3	1.5350 (18)	1.5371 (13)
P1—O1	1.5493 (18)	1.5502 (13)
P2—O5	1.5294 (18)	1.5253 (13)
P2—O4	1.5420 (18)	1.5444 (13)
		
*AlO_5_ trigonal bipyramid*		
Al—O2^ii^	1.7847 (19)	1.7731 (14)
Al—O1	1.8013 (19)	1.7908 (14)
Al—O5^iv^	1.8080 (18)	1.7979 (14)
Al—O3^iv^	1.8886 (19)	1.8818 (14)
Al—O4	1.9320 (18)	1.9271 (14)
		
*MnO_6_ octa­hedra*		
*M*—O6	2.0799 (19)	2.0052 (13)
*M*—O7	2.1990 (20)	2.0799 (15)
*M*—O4	2.2805 (18)	2.1512 (13)
O4⋯O4^i^	2.407 (5)	2.387 (4)
O6⋯O7	2.950 (3)	2.785 (2)
O6⋯O7^i^	2.962 (4)	2.844 (3)
O4⋯O6	3.192 (3)	3.065 (2)
O4⋯O7	3.254 (3)	3.089 (2)
O4⋯O7^i^	3.291 (3)	3.094 (3)
O6⋯O6^i^	3.372 (5)	3.132 (4)

Symmetry codes: (i) −*x*, *y*, −*z* + 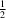 ; (ii) −*x* + 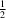 , *y* − 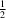 , −*z* + 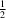 ; (iv) *x*, −*y* + 1, *z* − 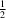 ; (vi) −*x* + 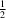 , *y* + 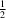 , −*z* + 1.

**Table 2:** Hydrogen-bond geometry (Å, °) for (I)

*D*—H⋯*A*	*D*—H	H⋯*A*	*D*⋯*A*	*D*—H⋯*A*
O7—H71⋯O1^i^	0.89	1.95	2.831 (3)	178
O7—H72⋯O3^ii^	0.87	2.04	2.897 (3)	166

Symmetry codes: (i) 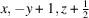 ; (ii) 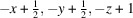 .

**Table 3:** Hydrogen-bond geometry (Å, °) for (II)

*D*—H⋯*A*	*D*—H	H⋯*A*	*D*⋯*A*	*D*—H⋯*A*
O7—H71⋯O1^i^	0.81	1.99	2.790 (2)	167
O7—H72⋯O3^ii^	0.86	2.11	2.961 (2)	170

Symmetry codes: (i) 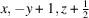 ; (ii) 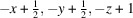 .

**Table 4:** Experimental details

	(NH_4_)MnAl_2_(PO_4_)_3_·2H_2_O	(NH_4_)NiAl_2_(PO_4_)_3_·2H_2_O
Crystal data		
*M*_r_	447.88	451.65
Crystal system, space group	Monoclinic, *C*2/*c*	Monoclinic, *C*2/*c*
Temperature (K)	298	298
*a*, *b*, *c* (Å)	13.3577 (7), 10.2279 (5), 8.7922 (5)	13.0711 (3), 10.1772 (2), 8.74476 (19)
β (°)	108.885 (6)	108.527 (3)
*V* (Å^3^)	1136.53 (11)	1103.00 (4)
*Z*	4	4
Radiation type	Mo *K*α	Mo *K*α
μ (mm^−1^)	1.85	2.44
Crystal size (mm)	0.05 × 0.04 × 0.02	0.11 × 0.04 × 0.03

Data collection		
Diffractometer	XtaLAB Synergy, Single source at offset/far, HyPix	XtaLAB Synergy, Single source at offset/far, HyPix
Absorption correction	Numerical (*CrysAlis PRO*; Rigaku OD, 2021 ▸)	Numerical (*CrysAlis PRO*; Rigaku OD, 2021 ▸)
*T*_min_, *T*_max_	0.938, 0.968	0.853, 0.962
No. of measured, independent and observed [*I* > 2σ(*I*)] reflections	5256, 1316, 1178	9320, 1328, 1281
*R*_int_	0.027	0.021
(sin θ/λ)_max_ (Å^−1^)	0.653	0.660

Refinement		
*R*[*F* ^2^ > 2σ(*F* ^2^)], *wR*(*F* ^2^), *S*	0.027, 0.077, 1.11	0.018, 0.056, 1.14
No. of reflections	1316	1328
No. of parameters	97	97
H-atom treatment	H-atom parameters constrained	H-atom parameters constrained
Δρ_max_, Δρ_min_ (e Å^−3^)	0.90, −0.51	0.42, −0.36

Computer programs: *CrysAlis PRO* 1.171.40.43a (Rigaku OD, 2021 ▸), *SHELXT2014/5* (Sheldrick, 2015*a* ▸), *SHELXL2016/6* (Sheldrick, 2015*b* ▸) and *VESTA* (Momma & Izumi, 2011 ▸).

## supplementary crystallographic information

### Ammonium manganese(II) dialuminium tris(phosphate) dihydrate (I) Crystal data

**Table:**

(NH_4_)MnAl_2_(PO_4_)_3_·2H_2_O	*F*(000) = 956.0
*M**_r_* = 447.88	*D*_x_ = 2.618 Mg m^−^^3^
Monoclinic, *C*2/*c*	Mo *K*α radiation, λ = 0.71073 Å
*a* = 13.3577 (7) Å	Cell parameters from 3620 reflections
*b* = 10.2279 (5) Å	θ = 3.9–27.6°
*c* = 8.7922 (5) Å	µ = 1.85 mm^−^^1^
β = 108.885 (6)°	*T* = 298 K
*V* = 1136.53 (11) Å^3^	Plate, colourless
*Z* = 4	0.05 × 0.04 × 0.02 mm

### Ammonium manganese(II) dialuminium tris(phosphate) dihydrate (I) Data collection

**Table:**

XtaLAB Synergy, Single source at offset/far, HyPix diffractometer	1316 independent reflections
Radiation source: micro-focus sealed X-ray tube, PhotonJet (Mo) X-ray Source	1178 reflections with *I* > 2σ(*I*)
Mirror monochromator	*R*_int_ = 0.027
Detector resolution: 10.0000 pixels mm^-1^	θ_max_ = 27.6°, θ_min_ = 3.2°
ω scans	*h* = −17→17
Absorption correction: numerical (CrysAlisPro; Rigaku OD, 2021)	*k* = −13→11
*T*_min_ = 0.938, *T*_max_ = 0.968	*l* = −11→11
5256 measured reflections	

### Ammonium manganese(II) dialuminium tris(phosphate) dihydrate (I) Refinement

**Table:**

Refinement on *F*^2^	Primary atom site location: dual
Least-squares matrix: full	Hydrogen site location: difference Fourier map
*R*[*F*^2^ > 2σ(*F*^2^)] = 0.027	H-atom parameters constrained
*wR*(*F*^2^) = 0.077	*w* = 1/[σ^2^(*F*_o_^2^) + (0.0358*P*)^2^ + 3.6714*P*] where *P* = (*F*_o_^2^ + 2*F*_c_^2^)/3
*S* = 1.11	(Δ/σ)_max_ < 0.001
1316 reflections	Δρ_max_ = 0.90 e Å^−^^3^
97 parameters	Δρ_min_ = −0.51 e Å^−^^3^
0 restraints	

### Ammonium manganese(II) dialuminium tris(phosphate) dihydrate (I) Special details

**Table:**

Geometry. All esds (except the esd in the dihedral angle between two l.s. planes) are estimated using the full covariance matrix. The cell esds are taken into account individually in the estimation of esds in distances, angles and torsion angles; correlations between esds in cell parameters are only used when they are defined by crystal symmetry. An approximate (isotropic) treatment of cell esds is used for estimating esds involving l.s. planes.

### Ammonium manganese(II) dialuminium tris(phosphate) dihydrate (I) Fractional atomic coordinates and isotropic or equivalent isotropic displacement parameters (Å^2^)

**Table:**

	*x*	*y*	*z*	*U*_iso_*/*U*_eq_	
Mn	0.000000	0.21386 (5)	0.250000	0.01325 (16)	
Al	0.16968 (5)	0.42298 (7)	0.07597 (8)	0.00506 (17)	
P1	0.29135 (5)	0.62403 (6)	0.33188 (7)	0.00855 (16)	
P2	0.000000	0.49748 (8)	0.250000	0.00678 (19)	
N1	0.500000	0.3634 (4)	0.250000	0.0357 (10)	
O1	0.20881 (14)	0.57892 (18)	0.1720 (2)	0.0129 (4)	
O2	0.27155 (14)	0.77104 (18)	0.3420 (2)	0.0130 (4)	
O3	0.27207 (14)	0.55150 (18)	0.4727 (2)	0.0132 (4)	
O4	0.07105 (14)	0.40324 (18)	0.1938 (2)	0.0118 (4)	
O5	0.06242 (14)	0.58669 (18)	0.3876 (2)	0.0116 (4)	
O6	0.09702 (14)	0.09481 (19)	0.1661 (2)	0.0179 (4)	
O7	0.11844 (17)	0.1969 (2)	0.4904 (3)	0.0262 (5)	
H71	0.148203	0.266954	0.546232	0.039*	
H72	0.160636	0.130057	0.499139	0.039*	

### Ammonium manganese(II) dialuminium tris(phosphate) dihydrate (I) Atomic displacement parameters (Å^2^)

**Table:**

	*U*^11^	*U*^22^	*U*^33^	*U*^12^	*U*^13^	*U*^23^
Mn	0.0131 (3)	0.0113 (3)	0.0168 (3)	0.000	0.0069 (2)	0.000
Al	0.0059 (3)	0.0047 (3)	0.0043 (3)	0.0007 (2)	0.0013 (3)	−0.0007 (2)
P1	0.0090 (3)	0.0086 (3)	0.0088 (3)	−0.0016 (2)	0.0039 (2)	−0.0006 (2)
P2	0.0071 (4)	0.0071 (4)	0.0063 (4)	0.000	0.0024 (3)	0.000
N1	0.041 (2)	0.0174 (19)	0.048 (3)	0.000	0.013 (2)	0.000
O1	0.0152 (9)	0.0132 (9)	0.0101 (8)	−0.0029 (7)	0.0039 (7)	−0.0037 (7)
O2	0.0168 (9)	0.0095 (9)	0.0137 (9)	−0.0030 (7)	0.0063 (7)	−0.0024 (7)
O3	0.0165 (9)	0.0124 (9)	0.0126 (9)	0.0010 (7)	0.0071 (7)	0.0029 (7)
O4	0.0125 (9)	0.0099 (8)	0.0153 (9)	0.0006 (7)	0.0078 (7)	0.0000 (7)
O5	0.0114 (8)	0.0123 (9)	0.0095 (8)	−0.0013 (7)	0.0010 (7)	−0.0036 (7)
O6	0.0107 (9)	0.0185 (10)	0.0265 (10)	−0.0019 (7)	0.0089 (8)	−0.0045 (8)
O7	0.0275 (12)	0.0201 (11)	0.0223 (10)	0.0016 (9)	−0.0040 (9)	−0.0044 (8)

### Ammonium manganese(II) dialuminium tris(phosphate) dihydrate (I) Geometric parameters (Å, º)

**Table:**

Mn—O6	2.0799 (19)	Al—O4	1.9320 (18)
Mn—O6^i^	2.0799 (19)	P1—O6^iv^	1.5152 (19)
Mn—O7	2.199 (2)	P1—O2	1.5342 (19)
Mn—O7^i^	2.199 (2)	P1—O3	1.5350 (18)
Mn—O4	2.2805 (18)	P1—O1	1.5493 (18)
Mn—O4^i^	2.2805 (18)	P2—O5	1.5294 (18)
Mn—P2	2.9008 (10)	P2—O5^i^	1.5294 (18)
Al—O2^ii^	1.7847 (19)	P2—O4	1.5420 (18)
Al—O1	1.8013 (19)	P2—O4^i^	1.5420 (18)
Al—O5^iii^	1.8080 (18)	O7—H71	0.8867
Al—O3^iii^	1.8886 (19)	O7—H72	0.8736
			
O6—Mn—O6^i^	108.33 (11)	O5^iii^—Al—O4	90.54 (8)
O6—Mn—O7	87.58 (8)	O3^iii^—Al—O4	176.17 (8)
O6^i^—Mn—O7	87.13 (8)	O6^iv^—P1—O2	112.34 (11)
O6—Mn—O7^i^	87.13 (8)	O6^iv^—P1—O3	108.44 (11)
O6^i^—Mn—O7^i^	87.58 (8)	O2—P1—O3	110.49 (10)
O7—Mn—O7^i^	170.96 (12)	O6^iv^—P1—O1	111.15 (11)
O6—Mn—O4	93.99 (7)	O2—P1—O1	105.01 (10)
O6^i^—Mn—O4	157.67 (7)	O3—P1—O1	109.38 (10)
O7—Mn—O4	93.15 (7)	O5—P2—O5^i^	106.74 (14)
O7^i^—Mn—O4	94.53 (7)	O5—P2—O4	113.09 (9)
O6—Mn—O4^i^	157.67 (7)	O5^i^—P2—O4	110.71 (9)
O6^i^—Mn—O4^i^	93.99 (7)	O5—P2—O4^i^	110.71 (9)
O7—Mn—O4^i^	94.53 (7)	O5^i^—P2—O4^i^	113.09 (9)
O7^i^—Mn—O4^i^	93.15 (7)	O4—P2—O4^i^	102.63 (14)
O4—Mn—O4^i^	63.72 (9)	O5—P2—Mn	126.63 (7)
O6—Mn—P2	125.84 (6)	O5^i^—P2—Mn	126.63 (7)
O6^i^—Mn—P2	125.84 (6)	O4—P2—Mn	51.31 (7)
O7—Mn—P2	94.52 (6)	O4^i^—P2—Mn	51.31 (7)
O7^i^—Mn—P2	94.52 (6)	P1—O1—Al	134.62 (12)
O4—Mn—P2	31.86 (4)	P1—O2—Al^iv^	144.61 (13)
O4^i^—Mn—P2	31.86 (4)	P1—O3—Al^v^	131.12 (11)
O2^ii^—Al—O1	123.95 (9)	P2—O4—Al	134.74 (11)
O2^ii^—Al—O5^iii^	115.92 (9)	P2—O4—Mn	96.83 (9)
O1—Al—O5^iii^	120.10 (9)	Al—O4—Mn	127.51 (9)
O2^ii^—Al—O3^iii^	91.29 (8)	P2—O5—Al^v^	139.42 (12)
O1—Al—O3^iii^	87.58 (8)	P1^ii^—O6—Mn	127.16 (12)
O5^iii^—Al—O3^iii^	92.86 (8)	Mn—O7—H71	121.5
O2^ii^—Al—O4	88.81 (8)	Mn—O7—H72	112.9
O1—Al—O4	89.19 (8)	H71—O7—H72	115.0

Symmetry codes: (i) −*x*, *y*, −*z*+1/2; (ii) −*x*+1/2, *y*−1/2, −*z*+1/2; (iii) *x*, −*y*+1, *z*−1/2; (iv) −*x*+1/2, *y*+1/2, −*z*+1/2; (v) *x*, −*y*+1, *z*+1/2.

### Ammonium manganese(II) dialuminium tris(phosphate) dihydrate (I) Hydrogen-bond geometry (Å, º)

**Table:**

*D*—H···*A*	*D*—H	H···*A*	*D*···*A*	*D*—H···*A*
O7—H71···O1^v^	0.89	1.95	2.831 (3)	178
O7—H72···O3^vi^	0.87	2.04	2.897 (3)	166

Symmetry codes: (v) *x*, −*y*+1, *z*+1/2; (vi) −*x*+1/2, −*y*+1/2, −*z*+1.

### Ammonium nickel(II) dialuminium tris(phosphate) dihydrate (II) Crystal data

**Table:**

(NH_4_)NiAl_2_(PO_4_)_3_·2H_2_O	*F*(000) = 904
*M**_r_* = 451.65	*D*_x_ = 2.720 Mg m^−^^3^
Monoclinic, *C*2/*c*	Mo *K*α radiation, λ = 0.71073 Å
*a* = 13.0711 (3) Å	Cell parameters from 14220 reflections
*b* = 10.1772 (2) Å	θ = 2.6–44.8°
*c* = 8.74476 (19) Å	µ = 2.44 mm^−^^1^
β = 108.527 (3)°	*T* = 298 K
*V* = 1103.00 (4) Å^3^	Plate, colourless
*Z* = 4	0.11 × 0.04 × 0.03 mm

### Ammonium nickel(II) dialuminium tris(phosphate) dihydrate (II) Data collection

**Table:**

XtaLAB Synergy, Single source at offset/far, HyPix diffractometer	1328 independent reflections
Radiation source: micro-focus sealed X-ray tube, PhotonJet (Mo) X-ray Source	1281 reflections with *I* > 2σ(*I*)
Mirror monochromator	*R*_int_ = 0.021
Detector resolution: 10.0000 pixels mm^-1^	θ_max_ = 28.0°, θ_min_ = 2.6°
ω scans	*h* = −17→17
Absorption correction: numerical (CrysAlisPro; Rigaku OD, 2021)	*k* = −13→13
*T*_min_ = 0.853, *T*_max_ = 0.962	*l* = −11→11
9320 measured reflections	

### Ammonium nickel(II) dialuminium tris(phosphate) dihydrate (II) Refinement

**Table:**

Refinement on *F*^2^	Primary atom site location: dual
Least-squares matrix: full	Hydrogen site location: difference Fourier map
*R*[*F*^2^ > 2σ(*F*^2^)] = 0.018	H-atom parameters constrained
*wR*(*F*^2^) = 0.056	*w* = 1/[σ^2^(*F*_o_^2^) + (0.0245*P*)^2^ + 3.4224*P*] where *P* = (*F*_o_^2^ + 2*F*_c_^2^)/3
*S* = 1.14	(Δ/σ)_max_ = 0.001
1328 reflections	Δρ_max_ = 0.42 e Å^−^^3^
97 parameters	Δρ_min_ = −0.36 e Å^−^^3^
0 restraints	

### Ammonium nickel(II) dialuminium tris(phosphate) dihydrate (II) Special details

**Table:**

Geometry. All esds (except the esd in the dihedral angle between two l.s. planes) are estimated using the full covariance matrix. The cell esds are taken into account individually in the estimation of esds in distances, angles and torsion angles; correlations between esds in cell parameters are only used when they are defined by crystal symmetry. An approximate (isotropic) treatment of cell esds is used for estimating esds involving l.s. planes.

### Ammonium nickel(II) dialuminium tris(phosphate) dihydrate (II) Fractional atomic coordinates and isotropic or equivalent isotropic displacement parameters (Å^2^)

**Table:**

	*x*	*y*	*z*	*U*_iso_*/*U*_eq_	
Ni	0.000000	0.22315 (3)	0.250000	0.00786 (10)	
Al	0.17218 (4)	0.42251 (5)	0.07491 (6)	0.00453 (12)	
P1	0.29302 (4)	0.62530 (5)	0.32906 (5)	0.00592 (11)	
P2	0.000000	0.49528 (6)	0.250000	0.00488 (13)	
N1	0.500000	0.3647 (3)	0.250000	0.0282 (7)	
O1	0.20866 (11)	0.57928 (13)	0.16956 (15)	0.0093 (3)	
O2	0.26781 (11)	0.77156 (13)	0.34168 (16)	0.0091 (3)	
O3	0.27630 (11)	0.54936 (13)	0.47120 (15)	0.0092 (3)	
O4	0.07228 (11)	0.39900 (13)	0.19411 (16)	0.0085 (3)	
O5	0.06302 (11)	0.58454 (13)	0.38790 (15)	0.0088 (3)	
O6	0.09292 (11)	0.10007 (14)	0.17281 (17)	0.0119 (3)	
O7	0.11014 (13)	0.20652 (15)	0.48072 (18)	0.0185 (3)	
H71	0.147718	0.262282	0.538535	0.028*	
H72	0.150318	0.137982	0.497836	0.028*	

### Ammonium nickel(II) dialuminium tris(phosphate) dihydrate (II) Atomic displacement parameters (Å^2^)

**Table:**

	*U*^11^	*U*^22^	*U*^33^	*U*^12^	*U*^13^	*U*^23^
Ni	0.00760 (16)	0.00725 (17)	0.00974 (17)	0.000	0.00416 (12)	0.000
Al	0.0051 (2)	0.0041 (2)	0.0044 (2)	0.00028 (18)	0.00137 (19)	0.00000 (17)
P1	0.0066 (2)	0.0055 (2)	0.0065 (2)	−0.00124 (15)	0.00321 (16)	−0.00082 (15)
P2	0.0047 (3)	0.0056 (3)	0.0045 (3)	0.000	0.0017 (2)	0.000
N1	0.0316 (16)	0.0160 (13)	0.0391 (17)	0.000	0.0143 (14)	0.000
O1	0.0120 (6)	0.0080 (6)	0.0078 (6)	−0.0016 (5)	0.0028 (5)	−0.0024 (5)
O2	0.0106 (6)	0.0065 (6)	0.0110 (6)	−0.0018 (5)	0.0044 (5)	−0.0021 (5)
O3	0.0111 (6)	0.0091 (6)	0.0090 (6)	0.0006 (5)	0.0056 (5)	0.0019 (5)
O4	0.0093 (6)	0.0073 (6)	0.0114 (6)	0.0005 (5)	0.0069 (5)	−0.0001 (5)
O5	0.0087 (6)	0.0097 (6)	0.0064 (6)	−0.0007 (5)	0.0002 (5)	−0.0020 (5)
O6	0.0085 (6)	0.0111 (6)	0.0183 (7)	−0.0002 (5)	0.0075 (5)	−0.0026 (5)
O7	0.0192 (8)	0.0153 (7)	0.0149 (7)	0.0010 (6)	−0.0032 (6)	−0.0033 (6)

### Ammonium nickel(II) dialuminium tris(phosphate) dihydrate (II) Geometric parameters (Å, º)

**Table:**

Ni—O6	2.0052 (13)	Al—O4	1.9271 (14)
Ni—O6^i^	2.0052 (13)	P1—O6^iv^	1.5180 (14)
Ni—O7^i^	2.0799 (15)	P1—O2	1.5361 (14)
Ni—O7	2.0799 (15)	P1—O3	1.5371 (13)
Ni—O4	2.1512 (13)	P1—O1	1.5502 (13)
Ni—O4^i^	2.1513 (13)	P2—O5^i^	1.5253 (13)
Ni—P2	2.7695 (7)	P2—O5	1.5253 (13)
Al—O2^ii^	1.7731 (14)	P2—O4	1.5444 (13)
Al—O1	1.7908 (14)	P2—O4^i^	1.5444 (13)
Al—O5^iii^	1.7979 (14)	O7—H71	0.8131
Al—O3^iii^	1.8818 (14)	O7—H72	0.8572
			
O6—Ni—O6^i^	102.68 (8)	O5^iii^—Al—O4	90.50 (6)
O6—Ni—O7^i^	85.94 (6)	O3^iii^—Al—O4	176.10 (6)
O6^i^—Ni—O7^i^	88.23 (6)	O6^iv^—P1—O2	113.48 (8)
O6—Ni—O7	88.23 (6)	O6^iv^—P1—O3	108.43 (8)
O6^i^—Ni—O7	85.94 (6)	O2—P1—O3	109.89 (8)
O7^i^—Ni—O7	170.66 (9)	O6^iv^—P1—O1	111.07 (8)
O6—Ni—O4	94.96 (5)	O2—P1—O1	104.47 (8)
O6^i^—Ni—O4	162.34 (5)	O3—P1—O1	109.41 (8)
O7^i^—Ni—O4	93.78 (6)	O5^i^—P2—O5	106.90 (11)
O7—Ni—O4	93.98 (6)	O5^i^—P2—O4	111.02 (7)
O6—Ni—O4^i^	162.34 (5)	O5—P2—O4	113.39 (7)
O6^i^—Ni—O4^i^	94.96 (5)	O5^i^—P2—O4^i^	113.39 (7)
O7^i^—Ni—O4^i^	93.98 (6)	O5—P2—O4^i^	111.02 (7)
O7—Ni—O4^i^	93.78 (6)	O4—P2—O4^i^	101.24 (10)
O4—Ni—O4^i^	67.41 (7)	O5^i^—P2—Ni	126.55 (5)
O6—Ni—P2	128.66 (4)	O5—P2—Ni	126.55 (5)
O6^i^—Ni—P2	128.66 (4)	O4—P2—Ni	50.62 (5)
O7^i^—Ni—P2	94.67 (4)	O4^i^—P2—Ni	50.62 (5)
O7—Ni—P2	94.67 (4)	P1—O1—Al	133.93 (9)
O4—Ni—P2	33.70 (3)	P1—O2—Al^iv^	142.39 (9)
O4^i^—Ni—P2	33.71 (3)	P1—O3—Al^v^	128.58 (8)
O2^ii^—Al—O1	124.32 (7)	P2—O4—Al	132.86 (8)
O2^ii^—Al—O5^iii^	117.33 (7)	P2—O4—Ni	95.68 (6)
O1—Al—O5^iii^	118.28 (7)	Al—O4—Ni	130.40 (7)
O2^ii^—Al—O3^iii^	92.12 (6)	P2—O5—Al^v^	140.21 (9)
O1—Al—O3^iii^	87.71 (6)	P1^ii^—O6—Ni	126.87 (8)
O5^iii^—Al—O3^iii^	93.10 (6)	Ni—O7—H71	129.9
O2^ii^—Al—O4	87.54 (6)	Ni—O7—H72	115.6
O1—Al—O4	89.27 (6)	H71—O7—H72	104.1

Symmetry codes: (i) −*x*, *y*, −*z*+1/2; (ii) −*x*+1/2, *y*−1/2, −*z*+1/2; (iii) *x*, −*y*+1, *z*−1/2; (iv) −*x*+1/2, *y*+1/2, −*z*+1/2; (v) *x*, −*y*+1, *z*+1/2.

### Ammonium nickel(II) dialuminium tris(phosphate) dihydrate (II) Hydrogen-bond geometry (Å, º)

**Table:**

*D*—H···*A*	*D*—H	H···*A*	*D*···*A*	*D*—H···*A*
O7—H71···O1^v^	0.81	1.99	2.790 (2)	167
O7—H72···O3^vi^	0.86	2.11	2.961 (2)	170

Symmetry codes: (v) *x*, −*y*+1, *z*+1/2; (vi) −*x*+1/2, −*y*+1/2, −*z*+1.
